# Cigarette Smoke Exposure Induces Neurocognitive Impairments and Neuropathological Changes in the Hippocampus

**DOI:** 10.3389/fnmol.2022.893083

**Published:** 2022-05-17

**Authors:** Aleksandar Dobric, Simone N. De Luca, Huei Jiunn Seow, Hao Wang, Kurt Brassington, Stanley M. H. Chan, Kevin Mou, Jonathan Erlich, Stella Liong, Stavros Selemidis, Sarah J. Spencer, Steven Bozinovski, Ross Vlahos

**Affiliations:** School of Health and Biomedical Sciences, RMIT University, Bundoora, VIC, Australia

**Keywords:** cigarette smoking, emphysema, neuroinflammation, microglia, cognition, synaptogenesis

## Abstract

**Background and Objective:**

Neurocognitive dysfunction is present in up to ∼61% of people with chronic obstructive pulmonary disease (COPD), with symptoms including learning and memory deficiencies, negatively impacting the quality of life of these individuals. As the mechanisms responsible for neurocognitive deficits in COPD remain unknown, we explored whether chronic cigarette smoke (CS) exposure causes neurocognitive dysfunction in mice and whether this is associated with neuroinflammation and an altered neuropathology.

**Methods:**

Male BALB/c mice were exposed to room air (sham) or CS (9 cigarettes/day, 5 days/week) for 24 weeks. After 23 weeks, mice underwent neurocognitive tests to assess working and spatial memory retention. At 24 weeks, mice were culled and lungs were collected and assessed for hallmark features of COPD. Serum was assessed for systemic inflammation and the hippocampus was collected for neuroinflammatory and structural analysis.

**Results:**

Chronic CS exposure impaired lung function as well as driving pulmonary inflammation, emphysema, and systemic inflammation. CS exposure impaired working memory retention, which was associated with a suppression in hippocampal microglial number, however, these microglia displayed a more activated morphology. CS-exposed mice showed changes in astrocyte density as well as a reduction in synaptophysin and dendritic spines in the hippocampus.

**Conclusion:**

We have developed an experimental model of COPD in mice that recapitulates the hallmark features of the human disease. The altered microglial/astrocytic profiles and alterations in the neuropathology within the hippocampus may explain the neurocognitive dysfunction observed during COPD.

## Introduction

Chronic obstructive pulmonary disease (COPD) is a significant health burden globally and it is currently the third leading cause of death worldwide (Vogelmeier et al., 2020). COPD is characterized by persistent respiratory symptoms and airflow limitations, decreasing the overall quality of life and increases the risk of mortality (Vogelmeier et al., 2020). COPD primarily arises due to the noxious particles and gases from chronic cigarette smoke (CS) which accounts for 95% of cases in industrialized countries (Caramori et al., 2016; Vogelmeier et al., 2020). Over time, CS propagates the oxidative stress burden and pro-inflammatory cell profile within the lungs leading to emphysema (Di Stefano et al., 1998; Hellermann et al., 2002; MacNee, 2005; Sharafkhaneh et al., 2008; Barnes, 2009; Brassington et al., 2021). It is hypothesized that inflammatory mediators and harmful oxidants “spill over” into the systemic circulation, resulting in other chronic comorbid conditions.

COPD-induced neurocognitive dysfunction is associated with reduced quality of life compared with the healthy age-equivalent population, with up to 61% of people experiencing these symptoms (Dodd et al., 2010; Dodd, 2015; Torres-Sánchez et al., 2015). It is reported that people with smoking history and COPD experience neurocognitive deficits, with impairments in working memory (Lv et al., 2020; Nadar et al., 2021), executive functioning (Dodd et al., 2013; Lv et al., 2020), attention (Paul et al., 2006), and delayed recall (Brunette et al., 2021). Moreover, Brunette et al. (2021) elegantly highlighted that people with COPD experiencing neurocognitive dysfunction have difficulties performing normal routine daily activities, further impairing their quality of life. Furthermore, people experiencing episodes of acute exacerbations of COPD have significantly worsened neurocognitive function when compared to people in a stable condition (Dodd et al., 2013; Crisan et al., 2014). Despite the known association between CS and impaired neurocognitive outcomes (Singh et al., 2014; Torres-Sánchez et al., 2015), the underlying mechanisms remain unknown. One possible mechanism could be microglia-dependent neuroinflammation. Under normal physiological conditions, microglia display a highly branched ramified morphology, surveying the microenvironment, modulating neurogenesis and synaptic plasticity, and subsequently influencing cognitive abilities (Nimmerjahn et al., 2005; De Luca et al., 2020a). Upon activation, microglial cells adopt an amoeboid morphology, releasing pro-inflammatory mediators and reactive oxygen species (ROS) (Lull and Block, 2010). However, persistent microglial activation can lead to neuronal and axonal loss (Lehnardt et al., 2003) orchestrating neurocognitive impairments.

We and others have previously shown that sub-chronic CS exposure in mice is sufficient to induce some of the key features of COPD including pulmonary inflammation and oxidative stress as well as comorbidities such as skeletal muscle wasting and vascular endothelial dysfunction (Botelho et al., 2010; Beckett et al., 2013; Chan et al., 2020; Brassington et al., 2021). However, longer durations of CS exposure (3–6 months) in mice are required to cause a decline in lung function, airspace enlargement (emphysema) and airway collagen deposition (Vlahos and Bozinovski, 2014). Our chronic CS exposure model in mice aims to recapitulate key pathological features seen in human disease such as impaired lung function, airway fibrosis, and emphysema. Moreover, there are limited studies investigating neurocognitive dysfunction in COPD and the underlying mechanisms responsible for this comorbidity. Thus, we aimed to investigate whether chronic CS exposure induces neurocognitive dysfunction like that seen in humans with COPD, and whether this is associated with a neuroinflammatory response within the hippocampus, a key region involved in memory formation.

## Materials and Methods

### Animals

All animal care and experimental procedures followed the ARRIVE Guidelines (Percie du Sert et al., 2020), Australian Code of Practice for the Care of Experimental Animals and RMIT University Animal Ethics Committee requirements (AEC #1521 and #1928). Seven-week-old male BALB/c mice (Animal Resource Centre Pty. Ltd., WA, Australia) were housed in micro-isolator cages at an ambient temperature of 21°C on a 12-h day/night cycle with *ad libitum* access to water and standard mouse diet.

### Cigarette Smoke Exposure

Mice were acclimatized for 1 week prior to experimental commencement. Mice were placed in an 18 L Perspex chamber in a standard fume hood cabinet and exposed to normal fume hood air (sham group; *n* = 34) or Winfield Red Cigarettes (total particulate matter of ∼419 mg m^–3^, ∼16 mg of tar, ∼1.2 mg of nicotine, and ∼15 mg of CO; Philip Morris, Melbourne, VIC, Australia; *n* = 34). CS-exposed mice were exposed to 9 full cigarettes/day, 5 days/week for 24 weeks. Mice were exposed to CS or room air three times a day, with a 2-h break between smoke sessions as described previously (Brassington et al., 2021). We have shown that this CS exposure regime provides similar carboxyhemoglobin levels to human smokers (Vlahos et al., 2006). Following 23 weeks of CS exposure (1 week prior to cull), mice underwent neurocognitive testing followed by lung function and tissue collection.

### Neurocognitive Assessment

#### Novel Object Recognition

**FIGURE 1:**
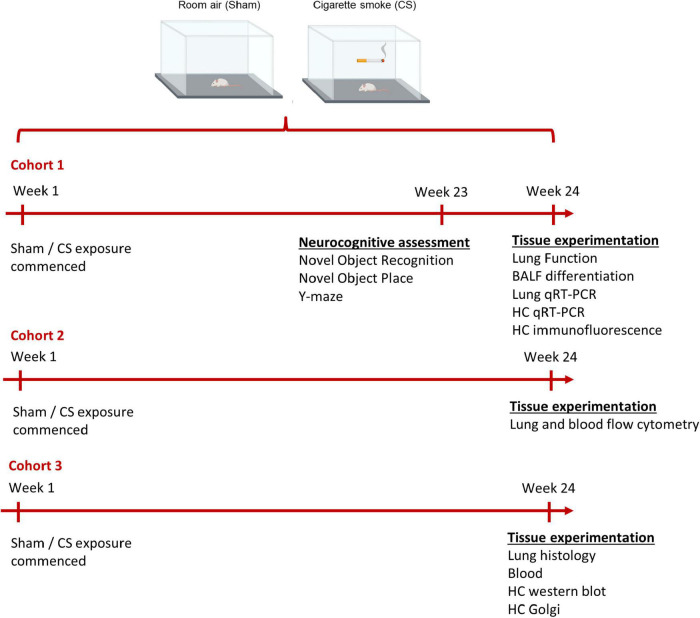
Timeline of experimental design. Mice were exposed to room air (sham) or cigarette smoke (CS; 9 cigarettes/day, 5 days/week) for 24 weeks. Cohort 1: neurocognitive assessments were performed on mice a week prior to protocol conclusion (week 23). Immediately prior to the cull, lung function assessment was performed (week 24). To examine the inflammatory profile within the lungs and hippocampus, we collected bronchoalveolar lavage (BAL) fluid for BAL differentiation and dissected the lungs and left hippocampi for qPCR. The right hippocampi was dissected for immunofluorescence. Cohort 2: lung and cardiac blood from a separate cohort of mice was collected for flow cytometric analysis. Cohort 3: a separate cohort was culled to examine lung pathology, blood brain barrier integrity (left hippocampi), and hippocampal dendritic spines (right hippocampi).

To examine whether CS exposure impaired working memory we performed the novel object recognition (NOR) task on mice from cohort 1 (Figure 1). To habituate mice to the arena, mice were allowed to explore the arena (dimensions 60 cm × 60 cm × 60 cm), twice, for 8 min each. The following day mice underwent the testing protocol which involved an 8-min acquisition phase with two identical objects, followed by a 1-h inter-trial-interval, and a retention phase for 8 min with one of the identical objects replaced with a novel object. The arena was thoroughly cleaned with 70% ethanol. Every session was recorded on a video camera and a double-blinded investigator assessed the exploration time of the objects divided by the time spent interacting with the objects. Each interaction involved sniffing and body contact with the objects, however, scoring did not occur when the mice leaned against, stood on, sat on, or faced away from the object. Results from the acquisition trial are expressed as total exploration of both objects and retention trial results are expressed as a Preference Index, calculated as the time spent with the novel object divided by the overall exploration time of the two objects in seconds multiplied by 100 [time_novel_ / (time_novel_ + time_familiar)_ × 100].

#### Novel Object Place

To assess spatial memory, we exposed the mice to the novel object placement (NOP) task (cohort 1; Figure 1). Similar to the NOR, the mice underwent a habituation and acquisition phase (as previously described). During the retention phase, one of the identical familiar objects was moved to novel location in the arena. Sessions were recorded on a video camera and assessed by a double-blinded investigator. The exploration time was scored by the amount of time spent interacting with the objects. Results are expressed as total exploration (acquisition phase) and Preference Index (retention phase; as previously described).

#### Spontaneous Alternation in the Y-Maze

To further assess spatial memory, we tested the mice in the spontaneous alternation in the Y-maze task (cohort 1; Figure 1). Mice were placed at the end of one arm of a symmetric Y maze (arm dimensions: 30 cm length × 11 cm width × 17 cm height) and were allowed to freely explore the arena for 5 min. All sessions were recorded with an overhead webcam and assessed by a double-blinded investigator. The series of arm entries were recorded, and an entry is defined as all four limbs passing the central area connecting the arms. An alternation was defined as the successive entry into three different arms during an overlapping triplet set (e.g., in the sequence 13231232, four alternations were recorded). Data are presented as the percentage of spontaneous alternation [number of alternations / (total number of arm entries − 2)]. After each session, the arena was cleaned with 70% ethanol. The total number of arm entries are presented as an index of ambulatory activity.

### Lung Function Testing

To assess pulmonary function, we performed lung function testing using a small-animal ventilator (FlexiVent system, Montreal, QC, Canada) in sham and CS-exposed mice (cohort 1; Figure 1; Wang et al., 2021). Briefly, mice were anaesthetized [125 mg/kg ketamine and 25 mg/kg xylazine intraperitoneal (i.p.)] and a tracheostomy was performed using an 18G cannula, with a suture tightening the wall of the trachea around the cannula. The mouse was subsequently connected to the FlexiVent system to assess different respiratory parameters. The inspiratory capacity (IC) was measured using a deep inflation maneuver. The quasi-static compliance (Cst) was produced from the pressure-volume (PV) loop maneuver, where PV curves and PV loop areas were generated. The forced vital capacity (FVC) was measured *via* a negative pressure-driven forced expirations (NPFE) maneuver similar to current preclinical literature (Giordano et al., 2019; Starkey et al., 2019), whereas in humans, FVC is driven by voluntary exhalation and air trapping occurs (Devos et al., 2017).

### Lung Collection and Histology

A separate cohort of mice were euthanized *via* i.p. injection by an overdose of sodium pentobarbital (Lethabarb; 240 mg/kg i.p.; Virbac, Sydney, NSW, Australia) to assess lung pathology (cohort 3; Figure 1). Lungs were fixed with neutral buffered formalin (NBF) solution *via* pressure-inflation techniques (Duan et al., 2012). Briefly, a gravitational pressure from 25 cm above bench level was used as NBF solution filled the lungs for 5 min. Following this period, lungs were excised and stored in NBF solution for 24 h, and then transferred to 70% ethanol. The left lobe of the lungs was sectioned into 4 μm sections and stained with hematoxylin and eosin (H&E) and Masson’s trichrome staining for collagen deposition (characterized by blue stains within the lung parenchyma) at the University of Melbourne Biomedical Sciences Histology Facility (Wang et al., 2021). Sections were imaged on an Olympus slide scanner VS120-SS (Olympus, Japan) at 20× magnification and 5 randomly selected fields within the distal region of the lung were analyzed. The mean linear intercept (L_m_) was quantified using a square grid (100 nm × 100 nm) which was created for analysis (VS120-SS). Using ImageJ, the alveolar surface intersecting with each of the horizontal grid lines were marked and distances between these alveolar surfaces was calculated whilst ensuring minimal amounts of blood vessels and airways were present. The L_m_ was calculated by adding the total distance and the amount of alveolar structures. Collagen deposits were measured within a drawn region of interest (ROI) where positively blue stained areas were present. The amount of collagen present (depicted as % area fraction) was assessed using cellSens Dimension*™* software (Olympus).

### Bronchoalveolar Lavage

Bronchoalveolar lavage (BAL) was performed *via* a surgical tracheotomy and lavaged *in situ* with 0.4 mL of chilled PBS, followed by further 0.3 mL aliquots of PBS until 1 mL of BAL fluid (BALF) was collected (cohort 1; Figure 1). Cytocentrifuge spots were created by centrifuging the BALF at 400 × *g* for 10 min. The cytospots were stained with Shandon Kwik-Diff Kit^®^ (ThermoFisher Scientific, Waltham, MA, United States) and Merck’s Hemacolor (eosin and thiazine solutions; Merck, Kenilworth, NJ, United States) as previously described (Brassington et al., 2021). Differential counts were performed, counting a total of 500 cells identifying macrophages, neutrophils, and lymphocytes using standard morphological criteria.

### Flow-Cytometry Analysis

A separate cohort of mice (cohort 2; Figure 1) were euthanized by an overdose of sodium pentobarbital (Lethabarb; 240 mg/kg i.p.; Virbac, Sydney, NSW, Australia). The left lobe of the lung was dissected and minced by using scissors and blood was collected *via* a cardiac puncture. Lungs were digested in an enzymatic digestion buffer (composition Liberase and Hanks balanced salt solution, Sigma-Aldrich, St-Louis, MO, United States) for 45 min with intermittent shakes to make a single-cell suspension. Single cell suspensions were filtered through a 40 μm strainer, and the reaction stopped by adding fluorescence-activated cell-sorting (FACS) buffer. Lung and blood suspensions were centrifuged at 400 × *g*, and red blood cells were lysed with ACK lysis buffer. Cell viability was determined by incubating with LIVE/DEAD Fixable Violet Dead Cell Stain Kit (ThermoFisher Scientific) for 15 min at 4°C. Cells were then washed with FACS buffer and centrifuged at 400 × *g* and incubated with cluster differentiation (CD)16/32 (2.4G2) to block Fc-mediated adherence of the antibodies. The white blood cells were stained with respective fluorescent labeled antibodies for flow cytometric analysis for 30 min at 4°C and washed twice with FACS buffer. The antibody panels used for staining, and in their different multicolor combinations were as follows: 1:500 Alexa Flour 700 anti-CD45 (30-F11); 1:500 APC anti-CD3 (145-2C11); 1:200 APC anti-CD4 (GK1.5); 1:1,000 PE-Cy7 anti-CD8 (53-6.7); 1:500 FITC anti-Ly6C (HK1.4); 1:500 APC-Cy7 anti-Ly6G (1A8); 1:500 BV421 anti-CD11b (M1/70); 1:200 FITC anti-NK1.1 (PK136); 1:200 PE-Cy7 anti-CD19 (B4); or live/dead Aqua (L34965). Cells were resuspended in FACS buffer and analyzed on the FACSAria (BD Biosciences, Franklin Lakes, NJ, United States) with FACSDiva software (BD Biosciences). Data analysis was performed using FlowJo software (Tree Star, Inc.). The cells are expressed as percentage of the CD45+ cells (live cells).

### Blood Collection and Counts

To identify possible systemic inflammatory responses as a result of chronic CS exposure, blood was collected *via* the inferior vena cava (cohort 3; Figure 1) and analyzed using the Cell Dyn Emerald Hematology Analyzer (Abbott Core Laboratory, Abbott Park, IL, United States). The blood was warmed to room temperature (RT) and mixed before 9.8 μL of blood was analyzed. Granulocytes, lymphocytes, and total leukocytes were counted *via* the Emerald Analyzer.

### Quantitative Real-Time PCR

Following BALF collection, mice were perfused with PBS and whole lungs and the left hippocampi were dissected and stored at −80°C until required (cohort 1; Figure 1). Lung tissue was crushed and approximately 10 mg was homogenized. Hippocampal samples were thawed and homogenized in 1 mL of TRIzol solution (ThermoFisher Scientific) using the TissueLyser LT^®^ (Qiagen, Valencia, CA, United States). Total RNA was extracted using the RNeasy^®^ Mini Kit (Qiagen). Isolated mRNA was then reverse transcribed to cDNA using a High-Capacity RNA-to-cDNA kit (ThermoFisher Scientific). Real-time PCR reactions were performed using Life Technologies pre-developed TaqMan assay reagents (Table 1) in triplicate using either the endogenous control *Rps18* (lung) or *Pgk1* (hippocampus) as the internal housekeeping control. We analyzed mRNA expression using the equation 2^–ΔΔC(t)^, where threshold cycle (C_t_) fluorescence is first detected significantly above background (Chan et al., 2020). Data are presented as fold expression relative to sham mice.

**TABLE 1:** TaqMan probes used for qPCR.

Target gene	NCBI ref seq	TaqMan assay ID	Product size
*Ribosomal protein s18 (Rps18)*	NM_011296.2	Mm02601777_g1	76
*Phosphoglycerate kinase 1 (Pgk1)*	NM_008828.3	Mm00435617_m1	137
*Tumor necrosis factor (Tnf)*	NM_013693.3	Mm00443258_m1	81
*Chemokine (C-C motif) ligand 2 (Ccl2)*	NM_011333.3	Mm00441242_m1	74
*Chemokine (C-X-C motif) ligand 1 (Cxcl1)*	NM_008176.3	Mm04207460_m1	111
*Matrix metallopeptidase 12 (Mmp12)*	NM_008605.3	Mm01168718_m1	138
*Glutathione peroxidase 1 (Gpx1)*	NM_008160.6	Mm00656767_g1	134
*NADPH oxidase 1 (Nox1)*	NM_172203.2	Mm00549170_m1	84
*Cytochrome b-245, beta polypeptide [Cybb (Nox2)]*	NM_007807.5	Mm01287743_m1	63
*Hypoxia inducible factor 1 alpha (Hif1*α)	NM_010431.2	Mm00468869_m1	75
*Integrin alpha M [Itgam (Cd11b)]*	NM_008401.2	Mm00434455_m1	69

### Blood–Brain Barrier Integrity

The right hippocampi from cohort 3 (Figure 1) was dissected and homogenized in RIPA lysis buffer. To determine the total amount of protein, a bicinchoninic acid (BCA) assay was performed. Samples were diluted in DEPC water to produce solutions containing 20 μg of protein, mixed with Laemmli sample buffer and heated to 95°C for 10 min. Proteins were separated by their molecular weights using gel electrophoresis (SDS-PAGE; 8–14% bis:acrylamide gel) and the membranes were transferred to methanol-activated polyvinylidene fluoride transfer membranes (Bio-Rad Laboratories Inc., Hercules, CA, United States) *via* the blotting sandwich technique. Membranes were dried and washed in blocking buffer (5% skim-milk based blocking buffer diluted in TBS-T), followed by incubation with primary antibodies overnight at 4°C [Albumin: 1:1,000, anti-chicken, Abcam (#ab106582). ZO-1: 1:1,000, anti-mouse, ThermoFisher Scientific (#33-9100). Occludin: 1:1,000, anti-rabbit, Cell Signaling Technologies (CST), Danvers, MA, United States (#91131s), β-actin: 1:1,000, anti-rabbit, CST, (#8457)]. Following the overnight incubation, the membranes were washed in TBS-T and incubated in HRP-linked secondary antibodies [Albumin: 1:3,000, anti-chicken IgG HRP-linked, Merck (#A9046). ZO-1: 1:3,000, anti-mouse IgG HRP-linked, CST (#7076s). Occludin and β-actin: 1:3,000, anti-rabbit IgG HRP-linked, CST (#7074s)] at RT for 1 h and prepared for chemiluminescence detection using the chemiluminescence Western Lighting Ultra Solution reagents (Perkin Elmer, Waltham, MA, United States). Detection of IgG did not utilize any primary antibody, however, followed the same procedure when blocking and administering the secondary antibodies. Band intensities were analyzed using the ImageLab software (Bio-Rad Laboratories Inc.) and normalized to β-actin housekeeping antibody.

### Immunohistochemistry

The right hemisphere of the brain was immersion-fixed in 4% paraformaldehyde solution for 24 h before cryoprotecting in 20% sucrose solution until further required (cohort 1; Figure 1). Brains were cut into 30 μm coronal sections using a cryostat (Leica Biosystems, Mt Waverly, VIC, Australia) and processed for immunofluorescent staining. Sections were stained for ionized calcium-binding adapter molecule-1 (Iba-1), glial fibrillary acidic protein (GFAP), neuronal nuclei (NeuN), doublecortin (DCX), and synaptophysin. Free-floating sections were washed with PBS-T and blocked with 3% BSA and 0.3% Triton X-100 (Iba-1 and GFAP) or 4% NHS, 3% BSA, and 0.3% Triton X-100 (NeuN, DCX and synaptophysin) for 2 h before submerging the sections in the primary antibody overnight at 4°C [Iba-1; 1:1,000, anti-rabbit, Wako Chemicals, Osaka, Japan (#1022-5). GFAP; 1:500, anti-rabbit, Dako, Bath, United Kingdom (#Z0334). NeuN; 1:1,000, anti-rabbit, Abcam, Cambridge, United Kingdom (#ab104225). DCX; 1:1,000, anti-guinea pig, Abcam (#ab2253). Synaptophysin; 1:2,000, anti-mouse, Sigma-Aldrich (#S5768)]. Following the primary incubation, the sections were transferred into the secondary antibody [Iba-1 and GFAP: 2 h, 1:500, Alexa Fluor 488 goat anti-rabbit, Life Technologies (#A-11008). NeuN: 2 h, 1:400, Alexa Fluor 594 goat anti-rabbit, Life Technologies (#A-11012). DCX: 2 h, 1:400, Alexa Fluor 488 goat anti-guinea pig Life Technologies (#A-11073). Synaptophysin: 2 h, 1:400, Alexa Fluor 488 rabbit anti-mouse, Life Technologies (#A-11001)]. Sections were counterstained with Fluoromount-G*™*, with DAPI (ThermoFisher Scientific) and imaged at 20× magnification on the Nikon Eclipse E600 microscope (Nikon, Minato City, Tokyo, Japan) and synaptophysin was imaged on the C1 confocal microscope (Nikon).

Microglia (Iba-1), astrocytes (GFAP) and mature neurons (NeuN) were quantified in five regions of the hippocampus including the CA1, CA3, and the hilus, subgranular (SG) and molecular regions of the dentate gyrus (DG) within a ROI, whilst immature neurons (DCX) and synaptophysin were quantified in the subgranular and hilus regions of the DG, respectively. These regions were identified through the Franklin and Paxinos Mouse Brain Atlas and were analyzed by an average of 3–4 sections per brain (between 1.22 and 2.70 mm caudal to the bregma). Microglial numbers were counted on ImageJ software [National Institute of Health (NIH), Bethesda, MD, United States] and the morphology of these cells were analyzed on Imaris software *via* sholl analysis (Bitplane, Oxford Instruments, Abingdon, United Kingdom). Microglial morphology was assessed in three cells per section and calculated using Imaris software as described previously (Spencer et al., 2019). Astrocyte density, NeuN and synaptophysin were analyzed using imaging analysis software cellSens Dimension*™* (Olympus, Tokyo, Japan) by thresholding the positive stains against the background in the thalamus region. Cells stained with DCX were manually counted in the SG region of the DG at 40× magnification.

### Golgi Staining and Neuronal Analysis

Dendritic spines on pyramidal neurons were differentiated using the FD Rapid GolgiStain*™* Kit (FD Neurotechnologies, Columbia, MD, United States) according to manufacturer’s instructions (cohort 3; Figure 1). We dissected 10 mm-thick brain slices that included the left hippocampi and the brain tissue was rapidly frozen in double distilled milliQ H_2_O and sectioned onto gelatin-coated slides at 100 μm using a cryostat. Sections were allowed to completely dry at RT in the dark and stained with FD solution and coverslipped with entelen mounting medium.

To analyze apical dendritic spines, images of the pyramidal neurons within the hippocampal CA1 were taken on an upright Olympus BX61 microscope (Olympus) at 100× magnification with a step-width of 0.15 μm using the z-stack option on the imaging software (cellSens Dimension*™* software, Olympus). For each mouse, three neurons were assessed across three different sections per animal, up to a total of five animals per group and images were analyzed on the Imaris software (Bitplane). To minimize variability in the analysis of neurons, we only include segments with unbroken secondary apical dendritic branches, the length of the branch was within 20–30 μm, and that the dendrite was not obstructed by other dendrites or background staining. Dendritic spines were assigned to six categories including Filopodia (length >2 μm), long thin (length <2 μm), thin (length <1 μm), stubby (length:width ratio <1), mushroom (length >0.6 μm; width >0.6 μm) and branched (consisting of 2 or more heads, regardless of length and width) (Risher et al., 2014; De Luca et al., 2020b). Data are expressed as the mean number of spines per 10 μm for each mouse.

### Data Analysis

All data are presented as the mean + standard error of the mean (SEM) unless otherwise stated; *n* represents the number of mice per treatment group. Statistical analyses performed between the sham and CS-exposed groups was *via* Student’s unpaired *t*-tests. For PV loop assessment, the data was analyzed with a repeated measures analysis of variance (ANOVA). The sholl analysis for microglial morphological assessment was grouped in bins of 5 μm and analyzed with a repeated measures ANOVA. All statistical analyses were performed using GraphPad Prism*™* software (Version 9). Statistical significance was assumed when *p* < 0.05.

## Results

### Chronic Cigarette Smoke Exposure Induces a Chronic Obstructive Pulmonary Disease Phenotype in Mice

**FIGURE 2:**
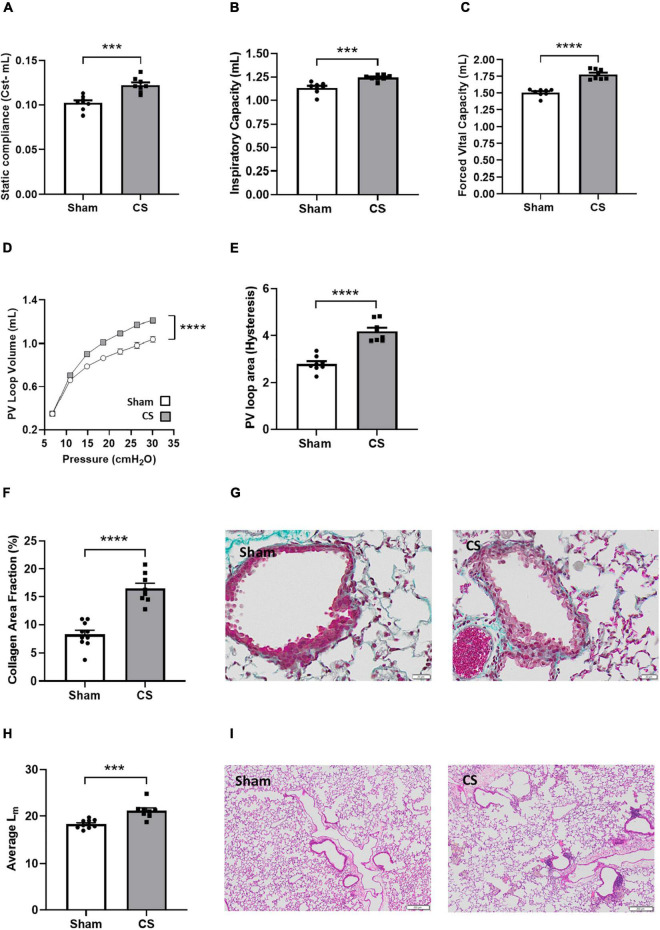
Chronic CS exposure worsened lung function and pathology. Mice were exposed to sham or CS (9 cigarettes/day, 5 days/week for 24 weeks). Lung function parameters assessed included **(A)** static compliance (Cst) (*n* = 8), **(B)** inspiratory capacity, **(C)** forced vital capacity, **(D)** pressure-volume (PV) loop, and **(E)** PV loop area. **(F)** Percentage area fraction of collagen (*n* = 8–10) and **(G)** representative images of collagen-stained (blue) sections. **(H)** Histological analysis of the mean linear intercept (L_m_) (*n* = 8–10) and **(I)** representative images of pressure-inflated lung sections. Data was analyzed by Student’s *t*-test and expressed as mean + SEM with significance being represented as; ***p < 0.001, ****p < 0.0001. PV loop data was analyzed *via* repeated measures and expressed as mean ± SEM with significance being represented as; ****p < 0.0001.

To examine whether chronic CS inhalation in mice recapitulates the human COPD phenotype, we assessed both lung function and lung pathology. CS-exposed mice displayed a significant increase in Cst compared to sham mice, demonstrating reduced elastic recoil due to the destruction of extracellular matrix and alveolar walls (*t*_(14)_ = 4.937, *p* = 0.0002; Figure 2A). The lungs of these mice were hyperinflated with increased IC (*t*_(14)_ = 4.678, *p* = 0.0004; Figure 2B). In NPFE maneuver, CS-exposed mice presented with a clear difference of 0.27 mL in FVC compared to shams (*t*_(14)_ = 8.183, *p* < 0.0001; Figure 2C). Weakened elastic properties and alveolar collapse in CS exposed mice were also supported by the upward shift in the PV loop curve (interaction of exposure by pressure: *F*_(6_,_84)_ = 64.50, *p* < 0.0001; Figure 2D) and the increased PV loop area (*t*_(14)_ = 7.188, *p* < 0.0001; Figure 2E). Airway fibrosis was also observed following CS exposure, with elevated levels of collagen deposited around the airways (*t*_(16)_ = 7.118, *p* < 0.0001; Figures 2F,G). CS-exposed mice displayed larger alveolar spacing within the lung parenchyma compared to shams (*t*_(16)_ = 4.574, *p* = 0.0003; Figures 2H,I).

**FIGURE 3:**
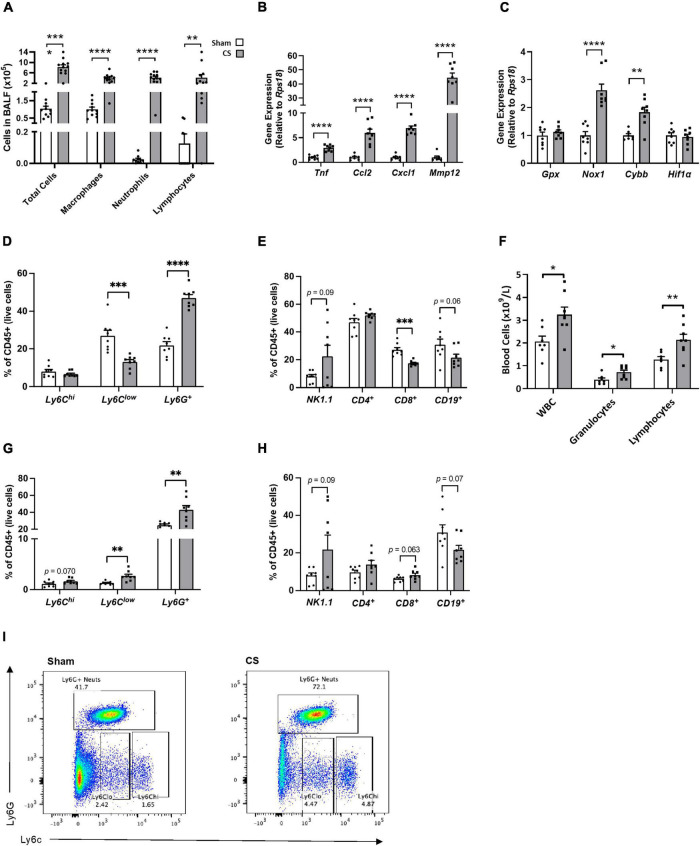
Chronic CS exposure causes lung and systemic inflammation. Mice were exposed to sham or CS (9 cigarettes/day, 5 days/week for 24 weeks). **(A)** Differential counts in the bronchoalveolar lavage fluid (BALF). **(B)** Inflammatory and protease mRNA expression in the lung. **(C)** Oxidative stress mRNA expression in the lung. **(D)** Percentage of innate immune cells in the lungs. **(E)** Percentage of adaptive immune cells in lungs. **(F)** Differential blood cell counts to assess leukocyte, granulocyte, and lymphocyte levels. **(G)** Percentage of innate immune cells in the blood. **(H)** Percentage of adaptive immune cells in blood. **(I)** Flow cytometric lung representative images. Data were analyzed by Student’s *t*-test and expressed as mean + SEM with significance being represented as; *p < 0.05, **p < 0.01, ***p < 0.001, and ****p < 0.0001. *n* = 7–8.

Cigarette smoke exposure caused an increase in BALF total cells, which was attributed to an increase in macrophages, neutrophils and lymphocytes compared to sham mice (Total number of cells: *t*_(21)_ = 7.700, *p* < 0.0001, macrophages: *t*_(21)_ = 6.044, *p* < 0.0001, neutrophils: *t*_(20)_ = 7.726, *p* < 0.0001, and lymphocytes: *t*_(21)_ = 3.195, *p* = 0.0044; Figure 3A). We also found a significant increase in cytokines, proteases and chemokines [tumor necrosis factor (*Tnf*)*: t*_(14)_ = 7.531, *p* < 0.0001; chemokine (C-C motif) ligand 2 (*Ccl2*): *t*_(13)_ = 5.778, *p* < 0.0001; chemokine (C-X-C motif) ligand 1 (*Cxcl1*): *t*_(14)_ = 11.67, *p* < 0.0001; matrix metallopeptidase 12 (*Mmp12*): *t*_(13)_ = 12.32, *p* < 0.0001; Figure 3B] and oxidative stress markers in the lungs [NADPH oxidase 1 (*Nox1*): *t*_(14)_ = 6.044, *p* < 0.0001. Cytochrome b-245 beta (Cybb): *t*_(13)_ = 4.122, *p* = 0.0012; Figure 3C]. There was a significant increase in the percentage of neutrophils [(CD11b^+^ Ly6C^+^) *t*_(14)_ = 9.308, *p* < 0.0001; Figures 3D,I] and a decrease in the percentage of patrolling monocytes [(CD11b^+^ Ly6C^low^) t_(14)_ = 4.391, *p* = 0.0006; Figures 3D,I] and cytotoxic T cells when compared to sham mice (CD3^+^ CD8^+^: *t*_(14)_ = 5.778, *p* < 0.0001; Figure 3E). CS exposure caused increased leukocyte levels within the blood, particular increases in granulocyte and lymphocyte numbers (leukocytes: *t*_(13)_ = 2.858; *p* = 0.135; granulocytes: *t*_(13)_ = 2.616; *p* = 0.0213; lymphocytes: *t*_(13)_ = 3.050; *p* = 0.0093; Figure 3F). Moreover, we saw a significant increase in patrolling monocytes, and neutrophils in the blood compared to sham mice *via* flow cytometric analysis [patrolling monocytes (CD11b^+^ Ly6C^low^): *t*_(14)_ = 3.703, *p* = 0.0024. neutrophils (CD11b^+^ Ly6C^+^): *t*_(14)_ = 3.583, *p* = 0.0030; Figures 3G,H].

### Cigarette Smoke Exposure Leads to Working Memory Impairment

**FIGURE 4:**
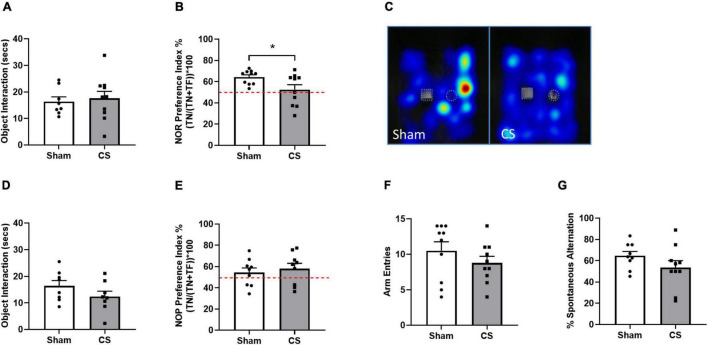
Chronic CS exposure decreased working memory in mice. Behavioral testing was performed on mice 1 week prior to protocol conclusion. **(A)** Total exploration time (seconds) in novel object recognition (NOR) acquisition phase. **(B)** NOR Preference Index. **(C)** Representative heatmaps illustrating familiar object (square) and novel object (circle) exploration in the NOR. **(D)** Total exploration time (seconds) in novel object placement (NOP) acquisition phase. **(E)** NOP Preference Index. **(F)** Arm entries in the Y Maze. **(G)** Percentage spontaneous alternation in Y Maze. Red dotted line in **(B,E)** represents a Preference Index of 50%. Data was analyzed by Student’s *t*-test and expressed as mean + SEM with significance being represented as *p < 0.05. *n* = 10 for the NOR and NOP and *n* = 14 for Y maze.

To understand the influence of CS exposure on neurocognitive function, mice underwent working (NOR) and spatial (NOP/spontaneous alternation Y maze test) memory testing. We observed no differences in object exploration in the NOR trial phase (*t*_(18)_ = 2.307, *p* = 0.0332; Figure 4A), however, CS-exposed mice were unable to differentiate between the novel and familiar object during the retention phase (Figures 4B,C), indicative of working memory impairments. However, spatial memory in the NOP remained intact following chronic CS exposure (Figures 4D,E) and we saw a downwards trend in the percentage of spontaneous alternation in the Y maze task (*p* = 0.07; Figures 4F,G) compared to sham mice.

### Cigarette Smoke Exposure Disrupts the Integrity of the Blood–Brain Barrier

**FIGURE 5:**
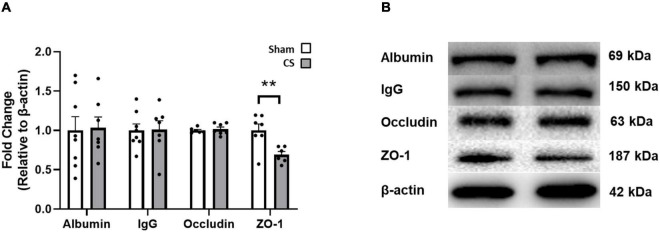
Chronic CS exposure decreased ZO-1 protein in the hippocampus. **(A)** Blood–brain barrier (BBB) protein levels in the hippocampus. **(B)** Representative Western blot images of BBB proteins. Data was analyzed by a Student’s *t*-test and expressed as mean + SEM with significance being represented as **p < 0.01. *n* = 7–8.

We performed immunoblotting to determine whether CS exposure disrupted key blood–brain barrier (BBB) proteins. The tight junctional protein ZO-1 was significantly decreased in CS-exposed mice compared to shams (ZO-1: *t*_(11)_ = 3.2, *p* = 0.0085; Figures 5A,B). However, occludin, albumin and IgG expression were unaltered in the hippocampus following CS exposure.

### Cigarette Smoke Exposure Induces Hippocampal Microglial Activation

**FIGURE 6:**
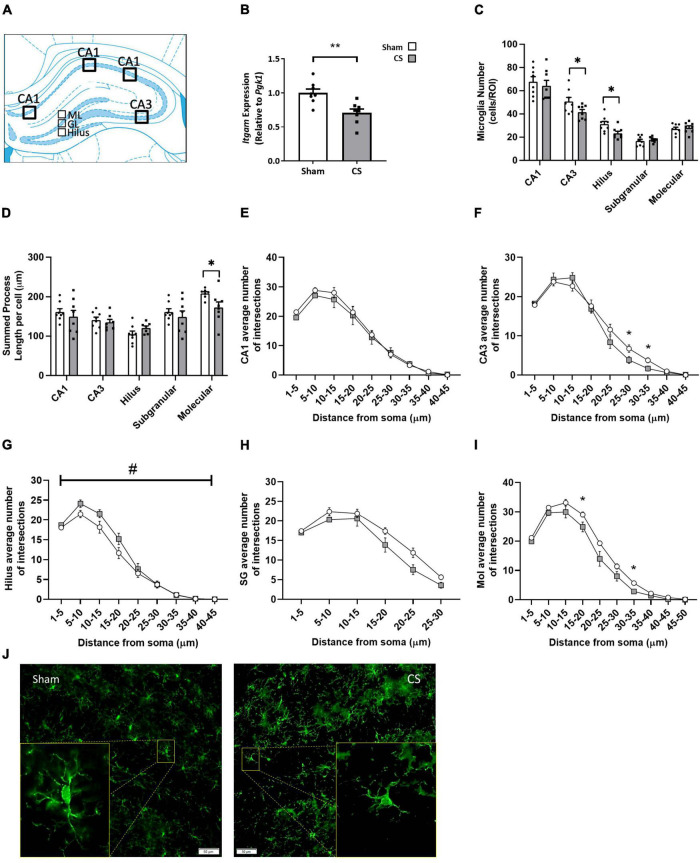
Chronic CS exposure decreased microglial numbers and increased microglial activation in the hippocampus. **(A)** Schematic diagram of the mouse hippocampus where regions of interest were analyzed. **(B)** Inflammation in the mouse hippocampi was assessed by qPCR. **(C)** Microglial (ionized calcium-binding adapter molecule 1 [Iba-1]-positive) numbers in the CA1, CA3, and the dentate gyrus [hilus, subgranular (SG), and molecular regions]. **(D)** Overall length of microglial processes in all regions of the hippocampus. **(E–I)** Sholl analysis of microglia in all regions of the hippocampus. **(J)** Representative images of Iba-1 analysis in the mouse hippocampus. qPCR and microglial number data were analyzed by Student’s *t*-test and expressed as mean + SEM with significance being represented as; *p < 0.05, **p < 0.01. Sholl analysis data was analyzed *via* repeated measures and expressed as mean ± SEM with significance being represented as *p < 0.05. The symbol “#” represents interaction between both independent variables when *p* < 0.05. *n* = 7–8.

To elucidate whether CS-induced working memory impairments was associated with neuroinflammation, we assessed inflammatory profiles within the hippocampus (Figure 6A). *Itgam* gene expression was decreased within the hippocampus in CS-exposed mice compared to shams, suggesting a reduction in CD11b-positive myeloid cells in this region (*t*_(14)_ = 3.698, *p* = 0.0024; Figure 6B). CS-exposed mice had fewer microglial numbers within the CA3 and the hilus (CA3: *t*_(14)_ = 2.193, *p* = 0.0457, hilus: *t*_(14)_ = 2.672, *p* = 0.0182; Figure 6C), with no observed differences in the CA1, SG and molecular regions. Chronic CS exposure significantly reduced the overall length of microglial processes in the molecular region of the hippocampus (*t*_(13)_ = 2.285, *p* = 0.0397; Figure 6D). Sholl analysis revealed a significant decrease in the average number of branching in the CA3 (*F*_(3_,_33)_ = 3.081, *p* = 0.045; Figures 6F,J) and molecular (*F*_(6_,_57)_ = 3.255, *p* = 0.009; Figures 6I,J) regions but no differences in the CA1 (Figures 6E,J) and SG (Figures 6H,J) regions compared to sham mice. In contrast, microglia in the hilus region were more branched compared to sham mice (*F*_(3_,_31)_ = 3.433, *p* = 0.028; Figures 6G,J).

### Cigarette Smoke Exposure Altered Hippocampal Astrocyte Profiles and Reduced the Number of Dendritic Spines

**FIGURE 7:**
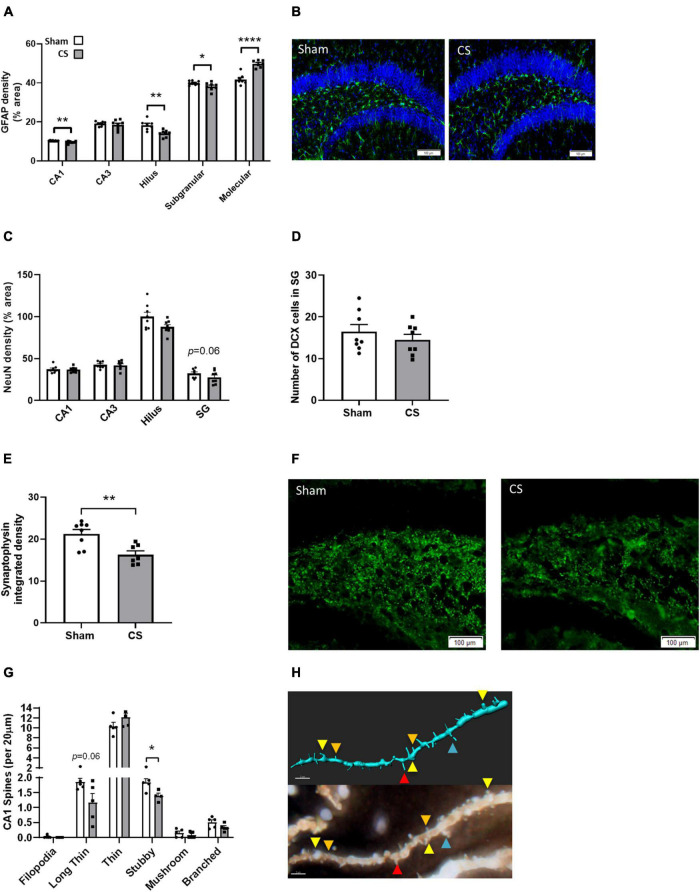
Chronic CS exposure decreased astrocyte numbers and altered synaptogenesis in the hippocampus. **(A)** Mouse hippocampi were stained with glial fibrillary acidic protein (GFAP) for the assessment of astrocytes in all hippocampal regions. **(C)** Mature neurons (NeuN-positive cells). **(D)** Immature neurons DCX-positive cells. **(E)** Synaptophysin staining in the hilus region of the dentate gyrus. **(G)** Golgi staining of secondary pyramidal neurons spines in the CA1 (*n* = 4–5). Representative images of **(B)** GFAP, **(F)** synaptophysin, and **(H)** golgi staining (red, long thin spine; orange, thin spine; yellow, stubby spine; blue, branched spine). Data was analyzed by Student’s *t*-test and expressed as mean + SEM with significance being represented as; *p < 0.05, **p < 0.01, ****p < 0.001. *n* = 7–8.

To elucidate whether CS-induced working memory impairments are associated with alterations in the neuronal profile, we assessed neurogenesis and synaptogenesis. CS exposure reduced astrocyte density in the CA1, hilus, and SG regions of the hippocampus (CA1: *t*_(13)_ = 3.349, *p* = 0.0052; hilus *t*_(13)_ = 3.928, *p* = 0.0017; SG: *t*_(14)_ = 2.203, *p* = 0.0448; Figures 7A,B) but an increase in density was observed in the molecular region (molecular: *t*_(13)_ = 6.974; *p* < 0.0001; Figure 7A) compared to sham mice. We identified no differences in NeuN, a mature neuronal marker (Figure 7C), or the immature neuronal marker, DCX (Figure 7D). Synaptophysin density was decreased in the hilus region following CS exposure (*t*_(14)_ = 2.911, *p* = 0.0114; Figures 7E,F). There was a significant reduction in the number of mature stubby spines in the secondary dendrites of CA1 pyramidal neurons (stubby: *t*_(7)_ = 2.969, *p* = 0.0208; Figures 7G,H) but no differences were observed in other spine types.

## Discussion

Our chronic CS exposure mouse model recapitulated both pulmonary and systemic features of human COPD. This was accompanied by neurocognitive impairments marked by alterations in working memory. Neurocognitive dysfunction was associated with the reduction of BBB tight junctional protein ZO-1, hippocampal neuroinflammation with microglial morphology indicative of a more-activated profile, despite there being fewer microglia in the region. Moreover, CS exposure suppressed hippocampal astrocyte density, reduced synaptophysin and stubby dendritic spine expression.

In conjunction with previously demonstrated lung inflammation, the current study demonstrated an emphysema phenotype alongside impairments in lung function. Altered lung compliance as seen in our CS-exposed mice was associated with more prominent airway spacing between alveolar structures due to alveolar destruction within the lung parenchyma, as well as increased expression of *Tnf*α and *Mmp12* (Chan et al., 2020). This replicates previous literature, where whole-body and nose-only CS exposure present with increase alveolar spacing in the lung tissue (Rinaldi et al., 2012; Shu et al., 2017). Additionally, we established a marked increase in collagen deposition within the lung parenchyma in CS-exposed mice like previous studies (Triantaphyllopoulos et al., 2011), which may inhibit proper repair of lung tissue, thus inducing irreversible lung damage, similar to that seen in people with COPD.

Recent literature has shown that people with COPD are more likely to develop neurocognitive impairments, either globally or in a single domain, with impairments in memory retention, information processing and executive function, than non-COPD smokers and healthy individuals (Dodd, 2015; Morris et al., 2019; Brunette et al., 2021). We have shown that 6 months of CS exposure in BALB/c mice is sufficient to recapitulate the lung profile of human COPD along with working, but not spatial, memory deficits. Similarly, Yang et al. (2019) showed that CS exposure for as little as 10 weeks is sufficient to induce spatial learning and memory impairments. We and others (Yang et al., 2019; Prasedya et al., 2020) have demonstrated that CS exposure induces working memory impairments, however, the mechanisms underlying neurocognitive comorbidities are still largely unknown.

Many studies have suggested an association between cognitive dysfunction and hypoxia-induced neurological damage (Gupta et al., 2013), airway obstruction (Cleutjens et al., 2014, 2017) as well as an elevation in inflammatory mediator expression (Duong et al., 1998). We demonstrate that the neurocognitive impairments following CS exposure may be associated with a loss of BBB integrity and increased neuroinflammatory response within the hippocampus compared to sham mice. Previous literature has shown that BBB endothelial cells and tight junction proteins are affected by pro-inflammatory cytokines following CS exposure (Prasad et al., 2015; Geng et al., 2018). Our study showed that CS-exposed mice had lower levels of the tight-junctional protein ZO-1 in the hippocampus which could contribute to brain damage and neurocognitive impairments. Upon activation, microglia produce pro-inflammatory cytokines and ROS, directly impacting endothelial cell tight junctions, reducing BBB integrity (Geng et al., 2018). Despite reduced astrocyte density within most regions of the hippocampus in our current cohort, CS-exposed mice showed an increase in astrocyte density in the molecular region, which may impact neurogenesis. Krzisch et al. (2015) have elegantly shown that astrocytes of the DG are expected to have greater structural plasticity to allow incorporation of new neurons. Thus, our CS-induced altered astrocyte density may not reduce the number of mature or immature neurons but may inhibit or halt the enseathing of afferent and efferent synapses of adult-born neurons (Krzisch et al., 2015). To access information and memories, a signal is relayed through the molecular region prior to traveling through the other hippocampal regions (Amaral et al., 2007; Roy et al., 2017). The increased astrocyte density in the molecular region of the DG could be assisting in the repair of the DG granule cells, or astrocytes could in fact be activated alongside microglia, potentially causing damage to these cells and hence, inhibiting normal neuronal function (Liddelow et al., 2017). Further investigation into the astrocyte profile is crucial in understanding the function of these cells.

Cigarette smoke-exposed mice displayed reduced expression of *Itgam* and microglial numbers within the hippocampus, as well as displaying a more activated morphology. A possible explanation could be that the noxious particles in CS are driving both microglial activation and/or a reduction in *Itgam* expression or cell death. In primary microglial cells, CS condensate induces apoptotic pathways alongside an activated and pro-inflammatory profile (Gao et al., 2014). Interestingly, a clinical study exploring the direct effect of smoking in non-COPD smokers showed decreases in the radiotracer (11C)DAA1106 [which binds to the microglial biomarker translocator proteins (TSPO)], compared to non-smokers (Brody et al., 2017). However, Hillmer et al. (2020) found no evidence of microglial activation in non-COPD smokers. These findings could be due to different radiotracer metabolism or bioavailability [(11C)DAA1106 and ^11^CPBR28, respectively], or the use of different timeframes (Hillmer et al., 2020). It is noteworthy that these studies only included non-COPD smoking participants, thus, caution must be taken when comparing to the current study. Previous pre-clinical literature assessing the effect of CS on the brain commonly only assessed pro-inflammatory profiles rather than investigating microglial profiles and were performed in whole brain tissue, rather than individual brain regions. It has been shown that as little as 3–6 weeks of CS exposure is sufficient to induce elevated expression of key pro-inflammatory cytokines in the brain (Khanna et al., 2013; Sivandzade et al., 2020), however, these studies do not assess the impact of CS exposure on neurocognition. Memory retention is relayed from the DG to the CA sections, proceeding from CA3 to CA1, then transferred to extra-hippocampal regions of the brain (Amaral et al., 2007). Thus, it could be speculated that the working memory impairments following chronic CS exposure, could be occurring due to altered microglial profiles within the hilus of the DG and the CA3 and specifically reducing microglial activation within these regions could improve CS-induced working memory impairments. Taken together, our current findings reinforce the concept that CS is likely to be responsible for the activation and modulation of microglial cells and this is associated with working memory impairments.

Microglia and astrocytes are integral in maintaining homeostatic neurogenesis, synaptogenesis and axonal development (Sofroniew and Vinters, 2010; Kohman and Rhodes, 2013). Our smoke exposure model induced an amoeboid microglial morphology, which may destabilize the oxidative equilibrium disrupting the neuronal profile within the hippocampus inducing neurocognition impairments. Although we found no differences in hippocampal neuronal populations following CS exposure, we showed a decrease in the pre-synaptic protein, synaptophysin, as well as a reduction in mature stubby spines in pyramidal neurons in the CA1 region, suggesting that CS exposed mice have weakened neuronal connections within the hippocampus. Ramified microglia modulate synaptogenesis to establish neuronal maturity, however, when chronically activated, these microglia may negatively impact the remodeling process. For example, elimination of microglia impaired efficient synaptic formation (Rogers et al., 2011), whilst another study showed ameliorating microglial activation improved working memory retention (Cope et al., 2018). Ho et al. (2012) demonstrated that CS exposure reduced synaptophysin expression and hence, a loss in synaptic integrity within the hilus region of the hippocampus. Overall, the apparent CS-induced increase in neuroinflammation, as well as reduced synaptophysin and mature dendritic spines are all consistent with the disruption of synaptic integrity, potentially orchestrating CS-induced neurocognitive impairments.

Neurocognitive comorbidities of COPD contribute to an increased mortality rate and an increased socioeconomic burden globally, however, there are no effective strategies treating these pathologies. In this study, we show a series of novel findings that CS-induced neurocognitive decline may be, in part, due to alterations in the hippocampal neuroinflammatory profile within the hippocampus. It will be imperative to evaluate whether pharmacologically targeting microglia-related molecular pathways could improve CS-induced cognitive outcomes. For instance, pharmacological inhibition of microglial activation using the tetracycline antibiotic, minocycline, has been shown to improve neurocognitive decline (Jiang et al., 2015; Cope et al., 2018). Moreover, stable COPD participants given a tetracycline analog showed improvements in lung function compared to vehicle-treated COPD participants (Dalvi et al., 2011). Therapeutics targeting oxidative stress may highlight potential targets as mice deficient in the antioxidant, Gpx-1, have elevated lung inflammation following CS exposure compared to wild-type CS-exposed mice (Duong et al., 2010; Oostwoud et al., 2016). Clinically, people with COPD have lower Nrf2 levels, a protein crucial to the upregulation of antioxidants and protection against oxidative stress (Fischer et al., 2015; Yamada et al., 2016). Thus, mitigating the pulmonary inflammation and the potential “spill-over” into the systemic and central circulation could halt neuroinflammation and the associated neurocognitive decline in CS-exposed mice. In-depth investigations of CS concomitant with pharmacological interventions targeting both pulmonary and central inflammation may represent an untapped therapeutic avenue for the development of COPD and the associated neurological comorbidities.

## Data Availability Statement

The original contributions presented in the study are included in the article/supplementary material, further inquiries can be directed to the corresponding author.

## Ethics Statement

The animal study was reviewed and approved by the RMIT University Animal Ethics Committee.

## Author Contributions

RV and SD: concept and design. AD, SD, HS, HW, KB, SC, KM, JE, and SL: acquisition of data. AD, SD, RV, HS, HW, KB, SC, KM, JE, SL, SS, SJS, and SB: data analysis and interpretation. SD, HS, KB, SC, and KM: technical assistance. All authors contributed to drafting, editing, and critical revision of the manuscript for intellectual content. RV provided all resources for the work and was the senior investigator, ensuring accuracy and integrity.

## Conflict of Interest

The authors declare that the research was conducted in the absence of any commercial or financial relationships that could be construed as a potential conflict of interest.

## Publisher’s Note

All claims expressed in this article are solely those of the authors and do not necessarily represent those of their affiliated organizations, or those of the publisher, the editors and the reviewers. Any product that may be evaluated in this article, or claim that may be made by its manufacturer, is not guaranteed or endorsed by the publisher.

## Abbreviations

AEC: Animal Ethics Committee
ANOVA: analysis of variance
BBB: blood–brain barrier
BAL: bronchoalveolar lavage
BALF: bronchoalveolar lavage fluid
BCA: bicinchoninic acid
*Ccl2*: chemokine (C-C motif) ligand 2
COPD: chronic obstructive pulmonary disease
CS: cigarette smoke
CD: cluster differentiation
*Cxcl1*: chemokine (C-X-C motif) ligand 1
*Cybb*: Cytochrome b-245 beta
DG: dentate gyrus
DCX: doublecortin
FACS: fluorescence-activated cell-sorting
FVC: forced vital capacity
GFAP: glial fibrillary acidic protein
*Gpx*: glutathione peroxidase 1
HC: hippocampus
H&E: hematoxylin and eosin
IC: inspiratory capacity
*Itgam*: Integrin alpha M
Iba-1: ionized calcium-binding adapter molecule-1
Lm: Mean linear intercept
*Mmp12*: matrix metallopeptidase 12
*Nox1*: NADPH oxidase 1
NPEE: negative pressure-driven forced expirations
NeuN: neuronal nuclei
NBF: neutral buffered formalin
NOP: novel object place
NOR: novel object recognition
*Pgk1*: phosphoglycerate kinase 1
ROS: reactive oxygen species
*Rps18*: ribosomal protein s18
RT: room temperature
SG: subgranular
TSPO: translocator proteins
*Tnf*: tumor necrosis factor.
